# Supplementary material for: A sum of its parts: A systematic review evaluating biopsychosocial and behavioral determinants of perinatal depression
Source: PLoS One. 2024 Jul 12;19(7):e0290059. doi: 10.1371/journal.pone.0290059 (PMC11244847; doi:10.1371/journal.pone.0290059)
Supplement: S1 Table — ®Study reported race and/or ethnicity. Author2 indicates the study was a secondary analysis. (DOCX) [file pone.0290059.s002.docx]

| **PREGNANCY** | | | | | |
| --- | --- | --- | --- | --- | --- |
| **First Author** | **Purpose/Aims** | **Risk of bias considerations** | | | |
|  |  | **Selection bias** | **Recall bias** | **Measurement bias** |  |
| Miyake^2^ (2022)  (N = 1744)  *Japan* | Examine the association between tryptophan intake and depressive symptoms during pregnancy in Japanese women. | Parent study is a prospective prebirth cohort study. The study is called Kyushu Okinawa Maternal and Child Health Study (KOMCHS). Same sample as Murakami et al. (2008) included in this review.  Specific to a large island in Southern Japan  Participation ranged from 5-39 weeks gestation. | None indicated beyond what is typically recognized with self-report measures:  CESD assesses past 7 days.  Diet History  Questionnaire  (DHQ) assesses past month. | *Timepoints: 5-39 weeks gestation*  An estimate of tryptophan intake was calculated based on an amino acid database developed in Japan. Tryptophan content is multiplied by each food the participant reports consuming, based on frequency and portion size. It is then summed and the sum is what is considered an estimate of tryptophan intake and used for analysis. Validity and reliability of this method of measurement is unclear, especially in perinatal populations where metabolic activity likely differs from that of the general population.  Participants mailed completed questionnaires to the data management center. Given the sensitive nature of the questionnaire responses (depression symptoms), unclear how methods for returning the questionnaires may have influenced item responses, if at all.  A cut-off score of ≥ 16 for CESD was used to define depression. The reference provided (Shima et al. 1985) was not available nor was the abstract of the study available. The other supporting reference (Radloff 1977) was not specific to the Japanese or perinatal population. Based on the references provided, unclear how applicable this instrument and the respective cut-off score are for measuring depression in pregnancy in a Japanese population.  ***The description of the DHQ and its use is nearly identical to the verbiage used to describe the DHQ in Murakami (2008) described in the perinatal section of this table. Upon further investigation, it appears Miyake and Murakami are or have been collaborators as evidenced by co-authorships, citations in one another’s publications, similarities in supporting references, and study method descriptions. Therefore, it is likely a number of biases gleaned from Murakami’s studies using the same sample may also be found in studies conducted by Miyake and vice versa. |  |
| ®Venkatesh^2^ (2019)  (N = 462)  *US* | 1) Determine whether antenatal depression was associated with two biomarkers of oxidative stress, 8-OHdG and 8-Isoprostane, and five biomarkers of inflammation.  2) assess whether the association between antenatal depression and spontaneous preterm birth was mediated by those biomarkers found to be significant in the primary aim. | Parent study was a prospective cohort study (LIFECODES birth cohort) at a women’s hospital in Boston, MA.  Unclear how 130 cases of preterm births from the birth cohort were selected or if that was simply all the preterm births within the cohort. Controls randomly selected.  Randomization process for controls not reported.  Considered to have prenatal depression (eligible) if participant was diagnosed with depression at any time during pregnancy per their electronic medical record. | Depression was not measured at any point during the study by study personnel, rather was determined by a diagnosis of depression at any time during pregnancy per their electronic medical record. | *Timepoints: Biospecimens ~ 10, 18, and 26 weeks gestations*  Urine and blood collected, specific times the samples were collected were not reported.  Cytokines were analyzed using Milliplex MAP High Sensitivity Human Cytokine Magnetic Bead Panel (EMD Millipore Corp); CRP measured using DuoSet enzyme-linked immunosorbent assay (ELISA) (R&D Systems); 8OHdG and 8-isoprostane were measured by Cayman Chemical. For 8-isoprostane, samples were eluted and then dried and resuspended in buffer before being measured with enzyme immunoassay (EIA)  Pre-term birth cases defined as spontaneous labor < 37 weeks gestation.  Though depression was the primary exposure variable, depression was not measured at any point during the study by study personnel, not reported what was used for prior diagnoses. |  |
| Chang (2018)  (N = 33)  *Taiwan* | Investigate if subjects with depression in pregnancy had higher levels of pro – inflammatory markers and lower levels of anti-inflammatory markers. | Case (n = 17); control (n = 16)  Conducted at a University hospital in the Obstetrics department where integrative care was provided (psychiatric team, obstetricians) to pregnant persons  Enrolled subjects from both second and third trimesters (between 16-28 weeks gestation).  All native Taiwanese subjects.  2 controls had a history of a psychiatric disorder.  No participants used psychotropic medications | None indicated beyond what is typically recognized with self-report measures:  Taiwanese version of the Mini-International Neuropsychiatric Interview (MINI) timeframes are consistent with diagnostic criteria in the DSM-IV or ICD-10  EPDS assesses past 7 days. | *Timepoints: 16-28 weeks gestation*  12 hr fasting blood samples were collected between 9a-10a.  CRP was measured by nephelometry, a latex particle-enhanced immunoassay (TBA-200FR) using a fully automatic biochemical analyzer (Unicel DxC 800 Synchron Clinical System); Cytokines were measured by Bio-Plex Suspension Array System 200 (Bio-Rad Laboratories) as well as a Procarta Immunoassay Kit using polystyrene beads and an appropriate diluent Plasma Standard Diluent Kit (Affymetrix-Panomics); fatty acid composition was analyzed by thin-layer chromatography and gas chromatography of methyl esters.  DSM-IV was used by a psychiatrist to determine depression cases.  Based on references provided to support the use of EPDS, unclear how appropriate the instrument is for use given study sample characteristics. For example:  Boyd et al. (2005), Teng et al. (2010), and Zubaran et al. (2010) were specific to the postpartum period and did not include Taiwanese women.  Chong et al. (2015) did not discuss psychometric properties of the instrument, rather the instrument was used in a study with a sample of women in Singapore and the specified cut-off scores (i.e., pregnancy ≥ 15, postpartum ≥13) were different than that in the present study (i.e., 12/13).  Su et al. (2007) suggest optimal cut-off scores are different between second and third trimester (i.e., 13/14, 12/13), yet the present study uses one cut-off score for both the second and third trimester with no supporting rationale. |  |
| ®Finy^2^ (2018)  (N = 214)  *US* | Examine the association between childhood abuse, low SES, and inflammatory markers during pregnancy | The parent study examined effects of flu vaccination on maternal and cord blood antibody levels in pregnant persons.  Participants were recruited primarily by faculty, staff, and students at The Ohio State University (OSU) and OSU Medical Center.  Enrollment gestational age spanned across all three trimesters (5-31 week).  Participants were excluded if they did not intend to deliver at the OSU medical center. | All variables besides Test of Negative Social Exchange (TENSE) and Childhood Trauma Questionnaire (CTQ) were measured ≤ 31 weeks gestation. TENSE and CTQ were measured ~30 days after at a follow up visit.  None other indicated beyond what is typically recognized with self-report measures:  Childhood Trauma Questionnaire (CTQ) asks adults to recall information about 5 types of childhood trauma (e.g., 3 types of abuse, 2 types of neglect) they may have experienced.  Test of Negative Social Exchange (TENSE) assessed past month.  Prenatal Life Events Scale (PLES) and Perceived Stress Scale (PSS) assesses the past year.  CESD assesses the past week. | *Timepoints: ≤ 31 weeks gestation or ~30 days after flu vaccination*  Blood collection times not reported.  CRP was measured using a solid-phase chemiluminescence immuometric assay with the IMMULITE 1000 Immunoassay System (Siemens Healthcare Diagnostics). IL-6 was measured by electrochemiluminescence in multiplex V-PLEX kits using a MESO QuickPlex SQ 120 Instrument (MesoScale Discovery).  The authors state CESD has been used in a number of empirical studies assessing depression in pregnancy and cite Nast et al. (2013) to support this statement. This single supporting reference is a systematic review on how to measure prenatal stress and positions depression as a source of stress. Further, this same referenced study reported, up to 2006, though the instrument has been used a total of 26 times, only one pregnant validation sample has been used. At the time of review no validity data was accessible to support its use in pregnant populations. Based on the references provided, unclear how appropriate the CESD is for use in pregnant sample such as that in the present study.  For the CTQ, only three of the five subscales were used (i.e., physical, sexual, and emotional abuse). Unclear why only three of the five were used in the analysis as the authors do not provide justification or a scientific rationale. |  |
| ®Miller^2^ (2018)  (N = 85)  *US* | To evaluate the association between psychotropic medication and maternal serum inflammatory biomarkers in women with antenatal depressive symptoms (ADS) in the mid-trimester. | Data obtained from the Measurement of Maternal Stress (MOMS) study which was a prospective multicenter observational study.  The sample were split into 3 groups. Group sizes were unequal (i.e., ADS responsive to treatment n = 16, ADS not responsive n =12, Untreated ADS n = 57).  Unclear rationale for investigating 12-21 weeks specifically. | None indicated beyond what is typically recognized with self-report measures:  CESD assesses the past week. | *Timepoints: 12-21 weeks gestation*  Biospecimen collection time not reported.  Cytokines were measured by electrochemoluminescent immunoassay on a MesoScale Discovery Instrument (SECTOR Imager 2400). CRP concentrations were measured using the Beckman Coulter UniCel DxC 800 System.  Based on references provided to support the use of CESD, unclear how appropriate the instrument is for use given study sample characteristics. For example:  Lewinsohn et al. (1997) was provided to support cut-off score of ≥ 23 for classifying untreated ADS or not responsive to medication, and <16 ADS for responsive to treatment. However, Lewinsohn and colleagues specifically looked at the efficacy of the instrument as a screener for depression in community-residing older adults (ages 50-96).  Authors reported they limited their regression models to a small panel of key covariates identified in the literature, but do not provide any references of said literature supporting the selection of 3 covariates.  Unclear what anti-depressant treatment participants were on and duration of treatment. The authors report assessing “any medications used for treatment of depression and/or anxiety” (p. 3). A number of the cited studies in support of the present study were specific to a particular class of antidepressant (i.e., SSRIs) or treatment itself (i.e., Paxil). Further, a number of the supporting references were specific to depression secondary to a specific condition or treatment of said condition (i.e., immunotherapy, psoriasis) making it unclear how these supported the present study given sample characteristics.. |  |
| ®Ross (2017)  (N = 90)  *US* | Examine the association between pregnant women’s close relationships and cytokine profiles in the third trimester. | Recruitment occurred at the Evanston Hospital’s Center for Maternal and Fetal Health and NorthShore Community Health Clinic  English speaking only  ≤ 25 weeks gestation at enrollment  Singleton pregnancy | None indicated beyond what is typically recognized with self-report measures:  EPDS assesses past 7 days.  Quality of Relationships Inventory (QRI) no specified timeframe.  Prenatal distress scale (PDS) assesses over the gestational period.  Perceived stress (PSS) assesses the past month. | *Timepoints: 22-26 and 32-36 weeks gestation*  Biospecimen collection time not reported.  Cytokines were measured using a MesoScale Discovery Multiplexing instrument (SECTOR Imager 2400) on a V-PLEX Proinflammatory Panel 1 (10-Plex) kit.  Sufficient information was provided for the QRI scale.  EPDS cut-off score not provided. Given EPDS scores were used as continuous variables rather than dichotomous, this did not pose issues for the analyses.  Only reference provided to support use of EPDS was Cox et al. 1987, which is the original article discussing the development and psychometric properties based on a postpartum sample in the United Kingdom with minimal sociodemographic information reported. Unclear based on references provided how appropriate this instrument was for use in the present sample.  The reference provided to support the PSS use in the present sample was the original article reporting the development of the PSS in a college student sample (mean age 20.75, SD = 4.41) and a smoking-cessation sample (mean age 38.4, SD = 11.57). The college student sample had more biological males than females, whereas the opposite was true for the smoking-cessation sample. Based on references provided it is unclear how appropriate the PSS is for use in pregnant populations. |  |
| ®Christian (2009)  (N = 60)  *US* | Examine associations among perceived stress, current depressive symptoms, and serum inflammatory markers among pregnant women from primarily lower socioeconomic backgrounds. | Recruited from Ohio State University Prenatal clinic.  Mean age 25 years  Study visit was completed on average at 15 weeks (SD = 7.8) gestation.  No other sampling information was provided. | None indicated beyond what is typically recognized with self-report measures:  CESD assesses depression symptoms over the past week.  Perceived stress (PSS) assesses the past month.  Interpersonal support evaluation list (ISEL) assesses social support but does not specify timeframe.  Test of Negative Social exchange (TENSE) assesses negative social experiences over the past month. | *Timepoints: First and second trimester*  Whole blood was collected between 9:30a – 1:30p.  Cytokines were assayed in duplicates using ultra-sensitive MultiPlex kits from MesoScale Discovery per manufacturer’s instructions.  Based on references provided it is unclear how appropriate CESD is for use given the sample characteristics. The one study referenced (Orr et al., 2002) was specific to perinatal populations; however, it was a study where CESD was used to measure depression symptoms to determine an association with spontaneous preterm birth and did not examine the psychometric properties of the instrument when used in pregnancy.  No CESD cut-off score was reported. Continuous scores were used rather than dichotomous, does not pose concern for analysis.  Two questions were asked to provide descriptive information about the perinatal person and partner’s happiness about the pregnancy on a scale of 1-10. Scores were summed and 1-3 indicated unhappiness, 4-7 ambivalence, and 8-10 happiness. It is unclear how these questions and the scoring were developed/determined given the reference did not specify a chapter or page number to refer to in the 300 page book. |  |
| **POSTPARTUM** | | | | | |
| **First Author** | **Purpose/Aims** | **Risk of bias considerations** | | | |
|  |  | **Selection bias** | **Recall bias** | **Measurement bias** | |
| Achytes (2022)  (N = 165)  *US* | Investigate whether a pro-inflammatory status in plasma, together with changes in the kynurenine pathway activity, is associated with the development of severe depression and suicidal behavior in the postpartum. | Case (n = 87), control (n = 60); an additional 18 participants displayed depression but did not fulfill the time criteria per the DSM-5 (i.e., pregnancy – 4 weeks postpartum). The 18 were not included in the primary analysis but were included in the sensitivity analysis.  Specific to participants with severe and suicidal depression  Diversity of sample sociodemographics unclear  Recruited from a Mother Baby program and OBGYN clinic. The mother baby program is a partial hospitalization program for those experiencing significant symptoms of depression and suicide. Women can bring their babies to the day program.  No explicit definition for “healthy controls” other than “psychiatrically healthy” due to not meeting formal DSM-5 criteria for PPD was reported.  Use of psychotropic medications were allowed due to severity of symptoms. Each class of medication is accounted for within the article. | None indicated beyond what is typically recognized with self-report measures:  SCID assesses past month as well as lifetime history.  EPDS assesses past 7 days.  Columbia Suicide Severity Rating Scale (C-SSRS) assesses past month or “since last visit.” | *Timepoints: 6-12 weeks postpartum*  Blood samples were collected between 9a-12p.  Cytokine assays were measured by the MesoScale Discovery platform and run, in duplicate, on a Sector 600.  Did not specify if measuring free and/or total tryptophan.  Tryptophan and kynurenine were analyzed using high-performance liquid chromatography with UV/Vi wavelength detection. Results were analyzed using the Chromeleon 7.2 Chromatography Data System (Thermo Scientific Dionex). Quinolinic acid was analyzed using gas chromatography-mass spectrometry. Ultra-high performance liquid chromatography and tandem mass spectrometry were used to determine plasma serotonin, kynurenic acid and nicotinic acid concentrations.  Minimal demographic factors measured. Limited to age, BMI, marital status, education, occupational status.  SCID was used to for the purposes of group allocation. Cases were those diagnosed with depressive episode with peripartum onset (PPD) per the formal DSM-5 criteria.  Did not report EPDS cut-off score yet classifies the level of depression for the present sample as moderate-severe. Unclear what score was used to classify a moderate-severe case. Reported EPDS mean was 18.5 (4.7).  C-SSRS was used to measure “active suicidal behavior,” and is defined as active preparations and actual suicide attempts. Such attempts can be completed, aborted, or interrupted. | |
| Dhiman^2^ (2021)  (N = 434)  *India* | Explore the association between vitamin B12 and probable PPD in South Indian population. | Parent study not described, states the study was conducted using archived plasma samples from a previous study. Cites study (Pillai et al., 2020)..  Recruited women who visited a tertiary care hospital at 6 weeks postpartum.  Specific to South of India population  Women with a history of mood disorders or antidepressant medication usage were excluded.  Case (n = 217), control (n = 217)  “Trained social workers used EPDS to screen for depression.” Given EPDS is a self-report questionnaire, it is unclear what is meant by the trained social workers used EPDS to screen for depression. | None indicated beyond what is typically recognized with self-report measures:  EPDS (English and Tamil versions) assesses past 7 days. | *Timepoints: 6 weeks postpartum*  Biospecimen collection time not reported.  Micronutrients were measured using commercially available ELISA kits.  No other methods related to biospecimen collection described.  Sociodemographic data and nutritional profile were recorded using a semi-structured questionnaire that was stated to be internally validated. No citations for the internal validation or questionnaire were provided.  Definition of postpartum depression: “an episode of depression within 6 weeks of delivery.” The reference the authors cite (WHO, 2010) appears to be International Classification of Diseases (ICD) codes but does not contain information that is consistent with the aforesaid definition and specified timeframe.  Based on references provided to support the use of EPDS and the designated cut-off score, unclear how appropriate instrument is for use given sample characteristics:  Benjamin et al. (2005) is referenced to support the use of the Tamil version of the EPDS and suggests a cut-off score of 8/9. However, Dhiman and colleagues state they use a different cut-off (i.e., ≥10) in their study as “suggested by experts from psychiatry department” because the study was conducted in a tertiary care center rather than a primary care center. Further, the supporting reference provided reports there are differences in scoring thresholds between the English and Tamil version. Unclear why two different versions with one cut-off score were used per references provided. | |
| Rihua^2^  (2018)  (N = 84)  *China* | To determine associations between PPD and plasma  neurotransmitters. | Parent study recruited from three hospital systems in China during their prenatal visit at 30-32 weeks gestation.  All participants were Han Chinese.  Participants with any recorded psychiatric history were excluded.  Cases (n = 42) and controls (n = 42) were randomly selected for biospecimen collection. Controls were matched by delivery hospital and age (±5 years). Ages ranged from 20-45 years.  Unclear methods of randomization. | None indicated beyond what is typically recognized with self-report measures:  EPDS (Chinese version) assesses past 7 days. | *Timepoint: 2 weeks postpartum*  Biospecimen collection time not reported.  High performance liquid chromatography with electrochemical detection was used to measure plasma levels of neurotransmitters except for neuropeptide Y and substance P which were measured using an enzyme-linked immunosorbent assay (ELISA).  No other methods for biospecimen collection or analysis were reported.  Postpartum depression is reported to occur within 4 weeks postpartum per DSM-5 peripartum specifier. In the present study, depression was assessed at 2 weeks postpartum at their routine follow up to reduce the burden on participants. It is common practice in this population for women to attend a 2-week postpartum follow up.  EPDS cut-off score ≥ 13 was used but differs from the cut-off score of ≥ 10 recommended by a validation study in a Chinese sample. Cox et al. (1987), the original article reporting the development of the instrument in a postpartum sample in the UK, was one of the references and does recommend ≥13 cut-off score. The additional reference (Lee et al., 1998) was a validation study of the Chinese version of the EPDS in a sample in Hong Kong. This reference suggested a cut-off score of ≥ 10. Unclear why a different cut off score of ≥ 13 was used when the validation study in a Chinese sample found the scales sensitivity to be only 0.41 which led the authors (Lee et al. 1998) to recommend ≥ 10 as the cut-off score in Chinese postpartum populations. Lowering the cut-off score to ≥10 doubled the scales sensitivity (.82), leading to the statement, “adopting the conventional cut-off of ≥13 for the Chinese version of the EPDS will yield an unsatisfactorily high rate of false negatives.” | |
| Veen  (2016)  (N = 42)  *Netherlands* | To investigate if alterations in tryptophan degradation in the postpartum period are associated with the occurrence of postpartum depression and postpartum psychosis. | Cases (n = 23), controls (n = 23)  Participants were recruited from a Mother Baby Inpatient Unit of a Psychiatric Medical Center if they had a severe postpartum onset of a psychiatric disorder. However, it is unclear how controls were recruited. If controls were recruited from the same unit, it is unclear how they were determined to be “healthy postpartum controls” given they were patients at a psychiatric medical center due to severe postpartum onset of a psychiatric disorder and exclusion criteria for controls was a psychiatric disorder.  Healthy controls were defined as EPDS ≤10, and were matched with cases based on ethnicity, method of delivery, and interval of blood collection.  Included participants with Major Depressive Disorder (n=11) and anxiety disorders with comorbid depression (n = 12). | None indicated beyond what is typically recognized with self-report measures:  EPDS assesses past 7 days.  SCID assesses past month as well as lifetime history. | *Timepoints: Postpartum timepoint(s) not specified.*  Serum samples collected between 8a-11a.  Tryptophan metabolites were analyzed using liquid chromatography with tandem mass spectrometry.  Did not specify if measuring free and/or total tryptophan.  A tryptophan breakdown index was calculated to estimate the activity of IDO and TDO enzymes. The single reference they have in the first paragraph under “analysis of tryptophan metabolites” provides evidence for methods of serum analysis. Though the authors explain how certain ratios reflect differing enzyme activity in paragraph two, the evidence to support the use of the calculation method of estimating enzyme activity is unclear as there are no references provided to support the use of said calculation.  Diagnosed according to DSM-IV-TR using the Structural Clinical Interview for Disease (SCID).  EPDS was used to assess depression cases along with the semi-structured interview, but EPDS was not used for any analyses. It was also used to determine the control group at the time of blood sampling. | |
| Comasco (2011)  (N = 272)  *Sweden* | Examine whether genetic variations in the monoaminergic neurotransmitter system, together with environmental stressors, contribute to the development of PPD symptoms | All women giving birth at a university hospital in Sweden were contacted by their midwife or midwife assistant after delivery and asked if they would like to volunteer to participate.  Excluded if not fluent in Swedish.  Excluded if offspring were immediately admitted to NICU.  Random sampling for blood samples of delivering mothers. | None indicated beyond what is typically recognized with self-report measures:  EPDS (Swedish version) assesses past 7 days.  Psychiatric contact was assessed over lifetime.  Stressful life events assessed over the last year.  Maternity stressors timeframe assessed unclear. | *Timepoints: 6 weeks postpartum, 6 months postpartum*  Blood sampling took place with routine intravenous catheterization before delivery.  Methods for processing assays for genotyping analyses not reported.  Unclear how “high” EPDS is defined as all that is provided is a cut-off score of ≥12 to identify PPD cases.  The authors reference Wicker and Hwang 1996 to support the specified cut-off score (≥12) as well as the validity of the instrument. It is stated the supporting study was “a large Swedish study,” and while the validation study had 1655 postpartum women complete the Swedish version of the EPDS at two timepoints (i.e., 2 and 3 months), it is important to note the results were based on 128 out of the 1655.  Psychiatric contact was measured by two questions: “Have you ever had contact with a psychiatrist or psychologist?” “If yes, can you specify the reason.”  Stressful life events scale was used to measure the number of significant life events occurring over the past year. The authors reference Rosengren et al. (1993) which is the developer of the scale but provide no other supporting literature for its use in other populations than the one in which it was developed (i.e., all men born in 1933). Life events were limited to serious illness of family member, serious concern about family member, death of family member, divorce or separation, forced to move house, forced to change job, been made redundant, feelings of insecurity at work, serious financial trouble, been legally prosecuted.  Maternity stressors were not measured by an existing instrument, rather, questions about maternity risk factors for PPD. However, how the authors determined which risk factors to measure is unclear. Those measured are: lack of breastfeeding, lack of sleep, lack of support from partner. | |
| Bailara (2006)  (N = 50)  *France* | Assess the correlation of intensity of baby blues, with the intensity of metabolic changes and brain tryptophan availability | Aside from 3 exclusion criteria, unclear how participants were selected upon hospital arrival.  Diversity of sample sociodemographics not reported. If sample characteristics are unclear, hard to determine generalizability. | None indicated beyond what is typically recognized with self-report measures.  Kennerley and Gath blues scale assesses within the past 10 days since delivery. | *Timepoints: “just before delivery” (baseline) and three days after delivery.*  Used Kennerley and Gath Blues Scale to measure postpartum blues.  Methods for biospecimen collection not reported.  Methods for biospecimen analyses were minimal; however, it was reported high performance liquid chromatography with electrochemical detection was used to measure both free and total tryptophan and serotonin whereas fluorometric detection was used for kynurenine and neopterin.  Brain tryptophan availability index (BTAI) was calculated per the Michaelis model for substrate competition on enzymes and transporters.  History of mood disorders were assessed with an abbreviated structured interview by clinicians using the MINI (version 5).  Citations provided to support all methods of measurement were sparse. | |
| Moses-Kolko^2^ (2008)  (N = 16)  *US* | To measure brain serotonin-1A (5HT1A) receptor binding potential (BP) in healthy and depressed postpartum women. | Parent study was exploring 5HT1A receptor status in postpartum depression.  Cases (n = 9), controls (n = 7); cases had either prenatal or postpartum depression. Five had unipolar depression, 4 had bipolar disorder. Controls had no personal history of a major Axis 1 disorder and no family history of a mood or psychotic disorder.  Excluded for MRI contraindications.  Excluded if had exposure to psychotropic or other medications that may alter cerebral physiology or monoamine function ≤ 3 weeks or ≤ 5 weeks for fluoxetine in depression cases. | None indicated beyond what is typically recognized with self-report measures:  SCID assesses past month as well as lifetime history.  EPDS assesses the past 7 days.  Hamilton rating scale (HAM_25_) assesses past week in comparison to patient reported baseline. | *Timepoints: ≤ 16 weeks postpartum*  Semi-structured interviews with Structured Clinical Interview for DSM-IV (SCID) and the 25-item Hamilton rating scale (HAM_25_) with a cut-off score of ≥14 were used to determine depression cases at enrollment only. Supporting evidence for the use of HAM_25_ and EPDS and the respective cut-off scores not provided. Given analysis uses scores as continuous rather than dichotomous, this does not pose issues for findings.  Reproductive hormones were characterized through menstrual cycle charting and morning serum concentrations of estradiol, progesterone, follicle stimulating hormone, luteinizing hormone, and prolactin on day of scan. Serum drawn between 9:45a-12:15p; mean blood draw time 10:24a (SD = 0:38).  PET scans conducted on an ECAT HR+ PET scanner (Siemens) in three-dimensional (3D) mode. For anatomical framework needed for analysis of PET data, MRI images were obtained using a 1.5 T Signa Scanner (GE Healthcare) as well as a 3-dimensional spoiled gradient recalled sequence. | |
| **PERINATAL** | | | | | |
| **First Author** | **Purpose/Aims** | **Risk of bias considerations** | | | |
|  |  | **Selection bias** | **Recall bias** | **Measurement bias** | |
| ®Sha  (2022)  (N = 114)  *US* | To determine whether cytokines and kynurenine metabolites can predict the development of depression in pregnancy. | Enrolled in first trimester and recruited from an Obstetrics and Gynecology clinic in Michigan.  Mean age 25 (±5.9)  60% of participants had a household annual income of ≤ $34,000  Authors consider the cohort to be “ethnically diverse” but only report race and do not report ethnicity. For the races reported: 47% were Caucasian, 26% were Black/African American, 10.5% were multiracial, and 1.8% identified as American Indian or Alaskan Indian. | None indicated beyond what is typically recognized with self-report measures:  EPDS assesses past 7 days.  SCID assesses timeframes consistent with the DSM-IV. | *Timepoints: “first trimester, second trimester, third trimester, postpartum.”*  Blood samples collected between 9A-12P.  Laboratory staff were blinded to all clinical parameters.  Did not specify if measuring free and/or total tryptophan.  Metabolites concentrations, except for quinolinate (QUIN) and picolinic acid (PIC), were measured using high-performance-liquid chromatography with UV/Vi wavelength detection. Results analyzed using Chromeleon 7.2 Chromatography Data System (Thermo Scientific). QUIN and PIC were separated by gas chromatography-mass spectrometry and quantified using Thermo Xcaliber and Waters Masslynx software.  Three metabolite ratios were created (i.e., kynurenine/tryptophan, QUIN/kynueric acid, and QUIN/PIC) to serve as proxies for enzymatic pathways.  Unclear when postpartum variables were measured.  The authors reference the Diagnostic Manual of Mental disorders (DSM-5) to support the use of the Structured Clinical Interview for DSM-IV (SCID) at baseline and reassessment for an EPDS score of >10 at any study visit. Sha specifies the use of SCID that was developed from criteria from DSM-IV but then references the newer version (DSM-5). Unclear from the present article how differences in diagnostic manual versions may influence the instrument, if at all.  Di Florio et al. 2017 and Rubeertsson et al. 2011 were referenced to support the use of EPDS in pregnant and postpartum persons and the respective cut-off score (≥ 13, or ≥10 for SCID reassessment). However, though Di Florio is postpartum specific and looking at different symptom expressions using EPDS, Rubertsson is specific to a study validating the Swedish version of EPDS in a pregnant sample. Based on these references alone, it is unclear how appropriate the EPDS and its respective cut-off scores are for use in the present sample. Further, there is no mention of SCID reassessment at EPDS >10, so the rationale for this process is unclear. | |
| ®Kimmel (2022)  (N = 30)  *US* | Analyze trajectories of serotonin and tryptophan-related metabolites, bile acid metabolites, and microbial composition related to psychiatric history and current symptoms across the perinatal period. | Restricted to singleton pregnancies.  Recruitment occurred through the University of North Carolina Obstetric and Women’s mood disorder clinics, a birth center, and campus advertising.  Diagnosis category for depression also included Premenstrual Dysphoric Disorder. | None indicated beyond what is typically recognized with self-report measures:  SCID assesses timeframes consistent with the DSM-IV.  EPDS assesses past 7 days.  The Generalized Anxiety disorder (GAD-7) instrument assesses past 14 days.  Automated Self-Administered 24-Hour Dietary Recall tool (ASA-24) assesses past 24 hours. | *Timepoints: Baseline - 1^st^ or 2^nd^ trimester, third trimester (32-37 weeks gestation), postpartum (5-10 weeks postpartum).*  Biospecimen collection time not reported for blood or fecal samples.  Did not specify if measuring free and/or total tryptophan.  Fecal samples used to assess gut microbiota.  Utilized a targeted approach for metabolite analysis. “Twenty of the most common bile acids in serum samples.” The authors stated three bile acids could not be analyzed reliably but no explanation was provided.  16S rRNA gene amplicon-sequencing data was carried out using the QIIME2 pipeline for microbial analysis of the fecal samples.  The title of the reference for the use of the SCID reports being the non-patient edition, so it is unclear how applicable its use is for perinatal populations with a potential mood disorder diagnosis.  The EPDS cut-off score used for classification of depression was not reported. However, EPDS was not used as a dichotomous variable, so it did not pose an issue for analysis.  Cox et al. (1987) was cited to support the use of the EPDS; however, this validation study was one conducted in the United Kingdom on a postpartum sample with minimal sociodemographic factors reported. Generalizability and appropriateness for use in the present sample is unclear based on the references provided.  The supporting reference for the use of the GAD-7 instrument was a validation study conducted in non-perinatal individuals. A majority of the participants were Non-Hispanic white (80%), 65% of the sample identified as female, and the mean age was 47.4 (15.5) years. Unclear how useful this tool is specific to perinatal populations, especially those of marginalized groups. | |
| Tebeka^2^ (2021)  (N = 3,252)  *France* | Assess the relationship between childhood trauma (CT) and perinatal depression, considering types of CT | Data obtained from the French Interaction of Gene and Environment of Depression in Postpartum Depression study (IGEDEPP) which is a large multicenter cohort study in Paris, France.  Specific to European populations who speak French.  Participants were excluded if they did not have French social insurance.  Unclear how recruitment occurred. Only states “women were included and assessed between the second and fifth day postpartum at a maternity department.”  A majority of participants were married, university educated, and employed. No other sociodemographics were provided. | Depression during pregnancy was assessed postpartum.  None other indicated beyond what is typically recognized with self-report measures:  French version of the Diagnostic Interview of Genetics Studies (DIGS) was used to measure depression.  The French version of the Childhood Trauma Questionnaire (CTQ) asks adults to answer questions about 5 types of childhood trauma (e.g., 3 types of abuse, 2 types of neglect). | *Timepoints: 2-5 days postpartum, follow up at 8 weeks and 1 year postpartum (depression in pregnancy was assessed postpartum)*  Psychiatric diagnoses were based on a semi-structured interview (DIGS) according to DSM-5 criteria at all three timepoints. The authors discuss the psychometric properties of the instrument, specifically, the French version. The semi-structured interviews were administered by trained psychologists and psychiatrists. Lifetime psychiatric history and suicide attempts were measured.  Depression during pregnancy was measured postpartum.  The CTQ cut-off score was used according to a validated study for the French version of the CTQ. | |
| Nazzari (2020)  (N = 97)  *Italy* | 1) Describe the cross-sectional and longitudinal association between tryptophan, kynurenine, and kynurenine/tryptophan ratio and depression symptoms in late pregnancy through the first year postpartum 2) examine the role of inflammatory (IL-6) and stress (cortisol) markers in moderating any associations 3) determine if specific to depressive symptoms or can be replicated with anxiety given high concurrence of these disorders | Specific to an Italian population.  Restricted to singleton pregnancies.  Not taking any current medications (including antidepressants).  Excluded for any psychiatric history other than depression and/or anxiety.  94.8% were middle-high SES. | None indicated beyond what is typically recognized with self-report measures:  SCID-I assesses timeframes consistent with the DSM-IV-TR.  EPDS assesses past 7 days.  The State Trait Anxiety Inventory subscale (STAI-S) instrument assesses anxiety in the present moment. | *Timepoints: biological markers were collected at 34-36 gestational weeks; other measures assessed “during pregnancy”, 2 days postpartum, and 3 and 12 months postpartum.*  Biospecimen samples were saliva for cortisol and plasma for inflammatory and tryptophan metabolites.  Only total tryptophan was considered. Authors state the lack of consideration of free tryptophan as a study limitation.  Between 34-36 weeks, six saliva samples were collected on 2 consecutive days at home (upon awakening, 30-min post-awakening, before going to bed) as well as blood “in a morning session” to measure inflammatory markers and tryptophan metabolism. These biological markers were measured again before the onset of labor (gestational age: *M = 39.48, SD = 1.25*). Unclear time of plasma sample collection other than “morning.”  IL-6 concentrations were assayed in duplicate using Quantikine High Sensitivity ELISA kits at LaboSpace in Milan per manufacturer’s protocol.  Serum tryptophan and kynurenine assays were executed at San Raffaele Scientific Institute, and quantified using a HPLC system with a fluorometric and UV-Vis detector.  EPDS cut-off >10 was used to determine depression. The supporting reference for EPDS use was Cox et at. (1987) which suggests a cut-off of ≥13 or ≥ 10 in a sample from the United Kingdom. Further, the reference, Benventuti et al. (1999), to support the use of the Italian version and the specified cut off score of the present study did not suggest the cut-off score used. Rather, the supporting reference suggested a ≥9 in an Italian sample, so it is unclear why >10 was used. Benventuti was also specific to postpartum sample, and the supporting reference they provided for its use in pregnancy (Murray & Cox, 1990) was conducted by the original developer of the EPDS (Cox) in a sample from the United Kingdom. Unclear how appropriate instrument is for use in pregnancy among an Italian sample based on references provided.  STAI-S cut-off score of 40 was used but sufficient support for this cut-off is not provided. One of the supporting references (Spielberger et al., 2012) was not accessible. An additional supporting reference (Meades & Ayers, 2011) is a review that does not include any studies with an Italian sample. For these reasons, it is unclear what the Spielberger study entailed and how these references support the use of the instrument in an Italian perinatal sample. It is also unclear how useful this instrument is in assessing perinatal anxiety given it may over or underinflate the occurrence of anxiety due to assessing anxiety at the time of assessment and does not ask about anxiety in preceding days, weeks, or months. Further, it is unclear if the instrument timeframe assessed was adapted or if it was used in its original form in the present study. | |
| Garman^2^ (2019)  (N = 384)  *South Africa* | Identify trajectories of perinatal depressive symptoms and their predictors among low-income South African women who were already at risk of depression during pregnancy. | The parent study was an RCT investigating the cost-effectiveness of a psychosocial intervention compared to enhanced usual care. Intervention was six counseling sessions that included elements of psycho-education. Enhanced usual care was monthly 5 min. phone calls for three months to assess health, suicide risk, and recent life events. Intervention was not reported to have an effect.  Recruitment for the parent study occurred in two community health centers located in Khayelitsha (South Africa) which is considered a low-income area.  Included participants were those who were considered “at risk” of depression. Those considered “at risk” had an EPDS score of ≥13.  isiXhosa speaking  Screened at first prenatal visit either in first or second trimester using EPDS. | None indicated beyond what is typically recognized with self-report measures:  EPDS (isiXhosa version) assesses past 7 days.  17-item Hamilton Depression Rating Scale (HDRS) is clinician rated as part of a semi-structured interview. Assessment is not specific to a particular time frame.  Mini International Neuropsychiatric Interview (MINI 6.0) assessed current and lifetime depression with timeframes consistent with diagnostic criteria.  World Health Organization Disability Assessment Schedule (WHODAS 2.0) assesses over the past 30 days.  Alcohol Use Disorder Identification Test (AUDIT) assesses frequency of consumption over the past week.  Multidimensional Scale of Perceived Social Support (MSPSS) does not have a specified timeframe.  The Household Food Insecurity Access Scale (HFIAS) assesses food insecurity over the past 30 days. | *Timepoints: Recruitment (first or second trimester), 8 months gestation, and 3 and 12 months postpartum.*  A cut-off score of 17 for HDRS was used to determine “clinically significant depressive symptoms.” The authors report, “a more structured HDRS was developed for the RCT for use by non-clinicians and this version was validated and showed good construct validity and internal consistency (Cronbach’s alpha = 0.74), inter-rater (0.97 to 0.98) and test-retest reliability (0.90) were excellent.” Further, the references they provide to support the use of this instrument had samples comprised of predominantly white insured women from the United States, except for the single study, conducted by three of the same authors from the present study, to validate the more structured HDRS for non-clinicians mentioned above. It is also important to note the validation study for the structured HDRS did not report a suggested cut-off score, so it is unclear if and how the structured HDRS cut-off score may differ from the clinician rated HDRS, if at all.  An EPDS score of ≥ 13 was used to screen for those “at risk” for depression. This cut off score is widely used in science and practice to suggest a person who is likely suffering from a depressive illness. Therefore, the rationale is unclear for classifying women “at risk” of depression with this instrument and cut-off score because the participants are not at risk if they actively have clinically significant depressive symptoms. Further, the reference stated to support its use (Lawrie et al. 1998) and respective cut-off score suggests a cut-off of ≥12 rather than the ≥13 cut-off that was used in the present study. Unclear why a different cut-off score was used based on references provided.  Mini International Neuropsychiatric Interview (MINI 6.0) was used to assess current depression and suicidality risk as well as a lifetime diagnosis of depression. High risk of suicide was considered with a score of ≥17. | |
| Teshigawara (2019)  (N = 132)  *Japan* | Investigate changes in plasma levels of tryptophan metabolites in pregnant women with depressive symptoms during pregnancy and/or the postpartum period | Recruited from those participating in a prenatal program at a University Hospital in Japan.  ≥ 20 years of age  Japanese speaking  Participants were divided into four groups per EPDS scores:  1. Non-depressive (ND), n = 62  2. Postpartum depressive (PD), n = 15  3. Temporary gestational depressive (TG), n = 22  4. Continuous depressive (CD), n = 33 | None indicated beyond what is typically recognized with self-report measures:  EPDS (Japanese version) assesses past 7 days. | *Timepoints: Pregnancy (≤ 25 wks gestation, ~36 wks gestation), 1 month after delivery.*  Biospecimen collection times not reported for plasma samples.  Did not specify if measuring free and/or total tryptophan.  High-performance liquid chromatography was used for measuring tryptophan and its metabolites.  EPDS cut-off score of ≥9 was supported by the provided reference. EPDS cut off scores for group allocation are as follows:   1. ND = ≤9 2. PD = ≥9 only at 1 month postpartum 3. TG = ≥9 only during pregnancy 4. CD = ≥9 during pregnancy and postpartum   Methods for group allocation may significantly limit findings for analyses specific to individual groups given groups vary in sample size, some of which are quite small (e.g., PD, TG).  The authors cite Ohoka et al. (2014) to support the use of EPDS in a Japanese population; however, the cited study is not a validation study rather a study using EPDS to measure the effects of maternal depression on infant mother attachment. Other references cited, Giri et al. (2015), Mori et al. (2017), Ohara et al. (2017, were also not validation studies or studies examining psychometric properties of the instrument rather were studies using the EPDS as an outcome measure in various Japanese populations, some of which did not have samples with persons identifying as women and all references but one were specific to the postpartum period. | |
| Vargas-Terrones (2017)  (N = 124)  *Spain* | Examine the effect of an exercise program during pregnancy on perinatal depression risk | Participants were recruited by obstetrician if they were <16 weeks pregnant with a singleton pregnancy in Madrid, Spain.  All control participants received usual care, whereas the intervention group participated in an exercise program designed for “heathy” pregnant women.  Exercise program took place in a fitness room in the hospital. Program was three sessions per week from 12-16 weeks gestation until the end of the third trimester (weeks 38-40).  There was a 69.3% adherence rate.  Though 124 were randomized with n = 70 being in the intervention group and n = 54 being in the control group, due to participant drop out or incomplete measures the week 38 analysis included: n = 70, n = 45 whereas the postpartum analysis included: n = 69, n = 47.  Epidat V.3.1 was used for randomization. | None indicated beyond what is typically recognized with self-report measures:  CES-D assesses depression symptoms over the past week. | *Timepoint: <16 weeks gestation, intervention 12-16 gestation through 38-40 weeks gestation, 6 weeks postpartum*  The CES-D was measured at three time points (12-16 weeks gestation, 38-39 weeks gestation, 6 weeks postpartum). A cut-off of ≥16 was used to indicate depression. Five studies were cited to support the use of CESD in pregnant populations. Of these five, four were RCTs using the CESD in their clinical trial examining the effects of exercise programs on pregnant persons and one was a RCT on postpartum persons. Two of the supporting articles had the same author. One was specific to obese and overweight persons, one was specific to an Egyptian population while another was specific to a Taiwanese population. Given a primary outcome is to examine how an exercise program effects postpartum depression risk, it is unclear why more support for use of the CESD was not provided for the postpartum period. Further, no studies examining the psychometric properties were mentioned so it is unclear how useful this tool and the specified cut-off score are in the present sample given the sample characteristics and references provided. | |
| ®Robertson Blackmore^2^ (2016)  (N = 171)  *US* | To examine the association between exposure to intimate partner violence (IPV) and IL-6 and TNF-a throughout the perinatal period. | Data obtained from a prospective, longitudinal cohort study.  Recruited from a hospital based obstetrical care practice serving predominantly low-income minority populations.  Ages 19-34, confirmed singleton pregnancy, <18 weeks gestation at enrollment, English speaking. | None indicated beyond what is typically recognized with self-report measures:  SCID assesses past month as well as lifetime history for depression, anxiety, and PTSD.  32 weeks gestation, EPDS was used to assess depressive symptoms over past 7 days. | *Timepoints: 18 and 32 weeks gestation (± 1 week), and 6 weeks and 6 months postpartum (± 1 week).*  Biospecimen collection times ranged from 8a-2p, but 14 samples were collected between 2-4P. Analyses with and without these 14 samples showed no difference, so data are reported based on the whole sample.  Women with a recent illness or fever were asked to come back a week later for biospecimen collection.  Clinical diagnosis of current and past history of depression and anxiety were made per DSM-IV-TR criteria from the mood disorder section of the SCID. While traumatic exposures other than IPV were determined per the PTSD section of the SCID. More specifically, a traumatic event was defined by meeting criterion A1 of the diagnostic criteria for PTSD from the DSM-IV-TR. Further, the one supporting reference for the use of SCID is for the “patient edition with psychotic screen.” Based on references provided, it is unclear why this particular version was selected and how appropriate for use this measure is for perinatal populations.  Group allocation to exposure versus non-exposure to IPV was determined by 5 questions about their history of IPV.  No EPDS cut-off score specified, but this did not pose issues for analysis because EPDS was not used as a dichotomous variable.  Only reference to support EPDS use was Cox et al (1987) which is the original article on the development of EPDS in a postpartum sample in the United Kingdom with minimal sociodemographic characteristics reported. | |
| Fasching (2012)  (N = 361)  *Germany* | Aim of the present study was to associate SNPs tagging genetic variation in *TPH2* with longitudinal measures of depressive symptoms in a cohort of pregnant women assessed in the third trimester of pregnancy, 2-3 days and 6 months after delivery. | German population  Caucasian only cohort  Gestational age at enrollment ≥ 31 weeks  Invited to participate when registering for upcoming labor and delivery.  Excluded those with pre-existing psychiatric disorders.  The present study is reported to be part of the Franconian Maternal Health Evaluation Study (FRAMES), but the genetic risk component was not added until recruitment was complete. Women were recalled for blood samples for the purposes of genetic analysis. Therefore, only one blood draw occurred at some point after study completion to collect the blood samples. | None indicated beyond what is typically recognized with self-report measures:  EPDS (German version) assesses past 7 days. | *Timepoints: Third trimester, 2-3 days postpartum, 6-8 months postpartum. Unknown when blood samples were collected other than postpartum.*  Single nucleotide polymorphisms (SNPs) were selected from the genotypes of the Caucasian CEU sample from Hapmap release II using tagger software implemented in Haploview version 4.2.  A commercially available DNA extraction kit, Puregene whole-blood DNA extraction, was used. TPH SNPs were analyzed on a Sequenom platform using iPlex technology in a multiplex assay using 10ng of DNA.  The German version of the EPDS was used to measure depressive symptoms at each timepoint.  No cut-off score was specified. The single supporting reference provided was specific to a postpartum sample who were predominantly married. The cut-off score threshold suggested by this reference was 9.5; however, scoring for EPDS results in an integer so it is unclear how a 9.5 was determined and what this meant for the present study. | |
| Lin^2^  (2009)  (N = 200)  *Taiwan* | To investigate the role of (tryptophan hydroxylase) *TPH2* in the etiology of peripartum major depression and anxiety disorder in a Han Chinese population in Taiwan. | Parent study was a longitudinal cohort that began in 1977 to investigate the causes and consequences of PPD. However, present study is cross-sectional.  Recruited from a University Hospital during a prenatal visit at 36 weeks gestation and followed up to the 8^th^ and 18^th^ weeks postpartum.  Specific to Han Chinese population  Those classified as cases (n = 117) were persons with peripartum (prenatal), postpartum, or perinatal depression, social and specific phobia, obsessive compulsive disorder, or generalized anxiety disorder. Controls (n = 83) were persons with no history of depression or anxiety. The two groups were age matched.  About half of the participants dropped out of the study because of the long follow-up time for phenotype assessment. | None indicated beyond what is typically recognized with the CM-SADS, SADS, and CM-SADS-C that have timeframes consistent with DSM-IV diagnostic criteria. | *Timepoints: Unclear for biospecimen collection. Appears mood disorder diagnosis was assessed at three time points (36 weeks gestation, and 8 and 18 weeks postpartum.*  Not clear when plasma samples were collected and if participants were rescreened for any potential psychiatric diagnoses at that time to determine the controls did not develop depressive symptoms. Methods for biospecimen collection were unclear. All that is reported is, “genomic DNA was prepared with approximately 10 ml of peripheral blood according to manufacturer’s protocols.” Kit used was Instagene whole-blood kit, BioRad laboratories.  Six SNPs were selected based on cited criteria and from an established profile of the human *TPH2* gene in Han Chinese. Two SNPs (T-473A and T-703G) were genotyped with direct sequencing in which polymerase chain reaction (PCR) was first used for amplification, whereas the other SNPs were genotyped with TaqMan assays using a sequence detecting system (ABI PRISM 9700HT; Applied Biosystems) per manufacturer’s protocol.  A Chinese modified version of the Schedule for Affective Disorders and Schizophrenia (CM-SADS) – lifetime and SADS – current version were used for group allocation (i.e., case, control) in accordance with the DSM-IV as well as the CM-SADS-C for follow up visits. Only one reference was cited to support the use of this measure in the present sample and is an article that reports on the initial scale development and reliability in a non-perinatal and non-Chinese population. Unclear how useful this instrument is in the present study given the references provided to support its use.  The present study appears to have aggregated participants with diagnoses at any timepoint within the study to classify them as cases. | |
| Murakami^2^ (2008)  (N = 865)  *Japan* | To examine the association between dietary GI and glycemic load (GL) in pregnancy and postpartum depression. | Data obtained from the Osaka Maternal and Child Health Study Group. This study was a prospective cohort study that investigated preventive and risk factors for maternal and child health outcomes.  Enrolled during pregnancy. When in pregnancy not specified. Follow up surveys were said to be collected between 2-9 months postpartum which is quite variable.  Sampled from Osaka Prefecture.  Participants mailed completed questionnaires to the data management center. Given the sensitive nature of the questionnaire responses (depression symptoms), unclear how methods for returning the questionnaires may have influenced item responses. | None indicated beyond what is typically recognized with self-report measures:  Diet history questionnaire (DHQ) assesses the past month.  EPDS (Japanese version) assesses past 7 days. | *Timepoints: Sometime in pregnancy and 2-9 months postpartum.*  An EPDS score of ≥9 was used to classify postpartum depression. The only references provided to support the utility of the instrument were two studies conducted by the original developers of the instrument, Cox et al. (1987). The instrument was developed in a non-diverse sample in the United Kingdom with suggested cut off scores of ≥13 or ≥10. Therefore, unclear why these studies were referenced given the present sample characteristics and differing cut-off scores. The one additional reference to support its use, translation into Japanese language, and respective cut-off score was specific to a postpartum sample, so it is unclear based on the references provided how useful the measure is in pregnancy.  The DHQ was used to assess dietary intake and calculate dietary factors. Calculations were conducted by using an ad hoc computer algorithm based on the Standard Tables of Food Composition in Japan. Dietary glycemic index (GI) was calculated by multiplying the contribution of each individual food to the daily intake of available carbohydrate by the foods GI value, then summed the products; dietary glycemic load (GL) was calculated by multiplying dietary GI by total daily available carbohydrate intake (divided by 100), based on a total of 72 major carbohydrate-containing foods. Crude values for dietary GI and total energy-adjusted values by density method (/4184 kJ) for dietary GL were used. References provided, author’s prior work, to support this method. One additional reference was provided; however, the sample of this validation study was in a non-perinatal population with participants who had mild hypercholesterolemia and were aged 38-69. The reproductive period alters metabolic activity to support the developing fetus. Unclear based on references provided how such methods account for metabolic changes in perinatal populations.  The authors consistently state their methods are described elsewhere requiring navigation to various sources to gather needed information to evaluate quality of methods applied. Author’s description of methods closely resembles the method descriptions in co-authored publications, such as, Miyake et al., (2022) included in this review. | |
