# Supplementary material for: A sum of its parts: A systematic review evaluating biopsychosocial and behavioral determinants of perinatal depression
Source: PLoS One. 2024 Jul 12;19(7):e0290059. doi: 10.1371/journal.pone.0290059 (PMC11244847; doi:10.1371/journal.pone.0290059)
Supplement: Attachment — Submitted filename: Response to reviewers_Table.docx [file pone.0290059.s003.docx]

**DUE DATE: Oct 23, 2023**

Revision table (Lines listed are those from the clean manuscript version)

| **#** | **Editorial embedded comments** | **Author revisions** | **Lines revised** |
| --- | --- | --- | --- |
|  | Formatting | |  |
|  | *Journal requirements*: Review and ensure manuscript meets PLOS ONE’s style requirements, including those for file naming. | We have modified our manuscript to ensure the manuscript meets the journal’s style requirements. |  |
| 1 | *Journal:* We note that you have included the phrase “data not shown.” This does not meet sharing requirements. | “Data not shown” was stated in **Table 2** referencing that one of the 25 articles included in the review, Comasco et al. (2011), did not report the statistical findings on the associations among genetic polymorphisms and postpartum depression at the 6-week and 6-month timepoints from their study. We have removed “data not shown” and stated, “However, Comasco and colleagues (2011) did not provide statistical output for these associations.” | **Table 2**  Row 10 |
|  | Introduction | |  |
| 2 | *R1:* The authors stated that “A majority of research on the etiology of perinatal depression has attempted to dissect it into two broad camps (i.e., internal factors, external factors) ” However, the authors repeatedly used the word "biological factors" later in the text rather than internal factors. If both refer to the same meaning, it is recommended that the authors use the same word to make it easier for the reader to understand or give examples of internal factors like external factors. | When revising the introductions, this sentence was removed. However, we agree terms need to be consistent, so we reviewed the manuscript to be sure biological is used rather than internal where appropriate. |  |
| 3 | *R1:* The authors stated that “In an era of team science, integrative approaches to investigation are not only feasible but desirable to address some of the world’s most complex health problems, like perinatal depression.” Please add references to support this statement. | Sentence modified and corresponding references were added [15, 16]. | 64-66 |
| 4 | *R1:* The authors do not mention whether any other studies have addressed similar issues. Therefore, the authors should mention whether there are similar studies currently and clarify what is added to this study compared to previous research. A clearer illustration of contribution or innovation should be further provided in the introduction. | Thank you, we found this comment to be particularly helpful in adding value to the manuscript. We have revised the **Introduction** and included the consensus of few studies that have aimed to address similar issues, and added how our study differs in terms of its contributions to the literature. Corresponding references included [13, 17] | 68-70; 79-80 |
|  | Methods | |  |
| 5 | *R1:* The authors stated that “Articles from any date were included if they focused on a timeframe within the perinatal period (i.e., conception-12 months postpartum), had participants that were 18 years or older, were available in the English language, investigated factors that belonged to at least one of the four domains (i.e., biological, behavioral, environmental, social), and had an outcome of depression or depression symptoms.” The literature search was limited to the English language, so the study might have overlooked similar studies published in other languages, which might be a limitation of this study. | Great point. This has been added as a study limitation under the new subheading **Limitations.** | 741-748 |
| 6 | *R1:* The author should provide the advantages of CASP and AXIS compared to other quality appraisal tools. | We added a description of our rationale for selecting CASP and AXIS over similar tools that are accessible. Corresponding references included [34, 35-36] | 140-150 |
|  | Results and discussion | |  |
| 7 | *R1:* The authors stated that “One article was excluded [32] during quality appraisal screening due to methodological concerns making the total articles included 25.” The author should specify the reasons for exclusion. | We stated eight reasons we were concerned about the quality of the study methods which collectively prompted exclusion. | 167-174 |
| 8 | *R1:* The sample sizes of the study ranged from 16 to 3,252. The conclusions drawn from the review may be affected by the low sample sizes of some studies. | We previously eluded to this when we discussed the differences in sample sizes (Lines 195-197). However, we agree this should be made more clear by explicitly stating it as a study limitation. We removed, what were Lines 195-197, and added a **Limitations** section where variability in sample sizes is one of the noted limitations. | 741-748 |
| 9 | *R2:* In the results, they put a lot of work on the Funding sources and the region (US and non-US based research), which, in my opinion, was not so valuable for the objectives of the review. This part may be shortened to make the review concentrate on the objectives. | Thank you for this insight. We refined and condensed the section **Contextual considerations.** | 351-386 |
| 10 | *R1*: The authors stated that “A total of four [33, 42–44] of the 25 studies discuss conducting a prior power analysis to calculate the needed sample size with half of those being US based studies [37, 42].” Please check if Ref. [33] is correctly cited. | We appreciate your attention to detail. Thank you for catching that this reference was not appropriately updated prior to submission. We double checked Ref. [33] and updated it accordingly [44]. We also re-checked all other references prior to submitting revisions to ensure accuracy. | 239 |
| 11 | *R1:* The authors stated that “Given every person develops within a maternal environment for up to 9.5 months,” Please add references to support this statement. | Sentence modified and corresponding references added [1, 64, 90-92]. | 383-384 |
| 12 | *R1:* “These demographic factors are important to consider because current evidence suggests those from lower SES and/or first-time mothers may be at increased risk of developing perinatal depression; however, there is conflicting evidence for education being a risk factor versus a protective factor.” Please add the corresponding references. In addition, the authors did not discuss the reasons for the contradictory results, please explain it. | We modified the sentence structure and provided potential reasons for contradictory findings. The corresponding references were added [13, 21, 23, 67]; [68]. | 203-208 |
| 13 | *R1:* “Conversely, it has been indicated that perinatal depression may result in early cessation or that difficulties with breastfeeding may contribute to perinatal depression symptoms.” Please add the corresponding references. | The two corresponding references were added [72, 73] | 230 |
| 14 | *R1:* “The bioavailability of essential amino acids (e.g., tryptophan, competitor amino acids), the precursors to a number of neurotransmitters commonly associated with psychiatric conditions, depends on dietary intake.” Please add references to support this statement. | Corresponding references added [24, 83]. | 324 |
| 15 | *R1:* “Conversely, it has been suggested that TRP has a higher affinity for the BBB than for albumin, and albumin bound TRP close to the BBB may separate from albumin to then transport across the BBB.” Please add references to support this statement. | Corresponding references added [22, 85, 86] | 331 |
| 16 | *R1:* The authors stated that “Moreover, IL-1β was found to be negatively associated with depression scores across four-time points (i.e., three trimesters, one postpartum time point).” However, the authors also stated that “Plasma IL-10 (anti-inflammatory cytokine) and IL-1β (pro-inflammatory cytokine) were not associated with increased risk for PPD.” These two findings may seem contradictory. Please explain it in a way that is easy for the readers to understand and add the corresponding references. | Revised paragraphs 2 and 3 under the ***Inflammatory markers and oxidative stress*** subheading to hopefully make these findings easier to understand. Corresponding references added [46, 54, 58]; [39]; [39, 42, 46, 50, 54, 58]; [42, 56, 58]; [39, 42]; [39, 42, 46, 50, 54, 56, 58]. | 415-434 |
| 17 | *R1:* “While sources of oxidative stress vary, evidence suggests the sources are largely related to environmental and lifestyle factors.” Please add references to support this statement. | Sentence modified and corresponding references added [93, 94]. | 437-439 |
| 18 | *R1:* “Additionally, though not a variable noted in any of the included studies, nausea and vomiting due to “morning sickness” or hyperemesis gravidarum (severe type of morning sickness) occurs in roughly 70% of pregnancies.” Please add references to support this data. | Corresponding reference added [118]. | 601 |
| 19 | *R1:* “The findings related to substance abuse, history of suicide attempts or current suicidal ideation, and exercise are consistent with existing literature” Please add the corresponding references. | Corresponding references added [7, 20, 119, 120]. | 613 |
| 20 | *R1:* The authors stated that “Interestingly, non-perinatal specific research that began examining the impact of COVID-19 on food insecurity found food insecurity to disproportionately impact racial and ethnic groups, and the states with the highest projected food insecurity rates based on overall population occurred in states that also have some of the highest maternal mortality rates (i.e., Louisiana, Texas)” However, this example does not seem to support the conclusion that food insecurity is a potential predictor of perinatal depression. Because in this example, the relationship between food insecurity and perinatal depression appears to be unclear. In addition, Please check if Ref. [76] is correctly cited. | Revised this paragraph to include findings specific to perinatal populations and removed prior example. Corresponding references were added [123-125]; [123, 124]. | 672-678 |
| 21 | *R1:* “Some evidence suggests the disruption of the serotonergic system is more prominent in biologically born females compared to males, and that the dysregulation of serotonin may partly explain why biologically born females experience depression at two times the rate of biologically born males.” Please add the corresponding references | Corresponding references added [128, 129]. | 691 |
|  | Comments not specific to any particular section | |  |
| 22 | *R2:* The participant characteristics could be included in the part of the social determinants. | We suspect that the ordering of the subheadings in the results section may have contributed to confusion. Thus, we changed the Participant characteristics subheading to **Description of study and sample characteristics** to make this more clear.  We separated **Tables 2-4** to be specific to each of the 4 domains (biological, behavioral, environmental social) so that the summary of findings are available for each domain under the respective subheading. | 176-231;  Table 2 – 394  Table 3 – 583  Table 4 - 632 |
| 23 | Prior to addressing the subsequent comments, we perceive it to be important to clarify how we interpreted the different terminology being used between the authors and Reviewer #2 for the primary outcome of our study (depression, emotion). | We defined perinatal depression in the third sentence of the **Introduction** (Lines 46-48), specified depression as our primary outcome of interest when detailing the purpose and aims of our study (Lines 85-89), and used “depression” throughout the entirety of the manuscript. For these reasons, there is confusion about the use of the term “emotions” rather than depression in the comments. Mental health clinicians and scientists recognize emotions (affect) as a subset of symptoms of depression and therefore do not use the terms interchangeably.  Since our study is specific to depression rather than a subset of symptoms (affect), we will address the comments based on the premise that “emotions” and depression are being viewed and used synonymously. If emotion and depression are not being used synonymously and it is suggested we focus on affective symptoms rather than the disorder as a whole, we do not perceive these comments to align with the specified objectives or scope of the study. |  |
| 24 | *R2:* And there are two other important things should be included/ discussed:  #1 - When the depression is determined is very important, since the emotion of the pregnant / postpartum women can vary much in the process. | As the perinatal period spans up to nearly 2 years, we agree that depression symptom severity will be highly variable across the perinatal period. We acknowledged this variability in **Tables 2-4**, which has findings separated by period (i.e., pregnancy, postpartum, perinatal). We also stated specific timepoints for each of the 25 studies in **Supplementary Table 1**. However, based on your feedback, it appears we can make this more clear. We have now modified **Tables 2-4** to include the timepoints in the design column.  Additionally, we have added a sentence under the **Limitations** subheading to state how timepoints assessed in this area of inquiry, as evidenced by our review, are highly variable which may challenge our ability to compare findings across studies. | Table 2 – 394  Table 3 – 583  Table 4 - 632  741-748 |
| 25 | *R2:* #2 - Another important thing, is the health of the fetus/ offspring, or pregnancy outcome, maternal emotion can be significantly affected by these. | We agree and current evidence supports that situational stressors (e.g., fetal/infant loss, admission to NICU) can be a primary cause of depression onset. For this reason, studies focused on depression in samples experiencing specific situational stressors were excluded from this review.  Our rationale for exclusion is as follows: when perinatal persons experience these situational stressors, the origin of depression onset is more straightforward as a primary contributing factor is readily identifiable (i.e., the acute situation). Since a primary contributor of depression onset is identifiable, the ability to detect and therefore intervene are typically more straightforward and are often specific to a subpopulation. For example, when depression onset occurs as a result of a stillbirth (acute situation), the primary contributor to depression onset is largely a result of the situation (stillbirth). Therefore, the interventions warranted to treat the depression are also more straightforward as they will be similar to that of a very specific subpopulation of individuals who’s root cause of depression onset was a situational factor (e.g., stillbirth).  Our study aimed to advance knowledge on determinants and interactions among determinants that may contribute to depression risk and onset in perinatal persons. Thus, we perceived including studies focused specifically on subpopulations experiencing situational stressors known to contribute to depression onset to have potential to convolute the results.  To provide clarity for the readers on this exclusion  criteria, we revised the last paragraph under the  **Inclusion and exclusion criteria** subheading to  make this more clear and state our rationale for  this exclusion criteria. Corresponding references  added [33, 34] | 122-127 |
